# Effect of closed reduction with external fixation versus open reduction and locking plate internal fixation on postoperative wrist function in elderly patients with distal radius fractures

**DOI:** 10.1097/MD.0000000000049683

**Published:** 2026-07-17

**Authors:** Weidong Chen, Yu Nie, Naxin Zhou

**Affiliations:** a Orthopedic Department, China Three Gorges University, Yichang Cent Peoples Hospital, Yichang City, Hubei Province, China.

**Keywords:** closed reduction, distal radius fracture, elderly, external fixation, locking plate, wrist function

## Abstract

Distal radius fractures are common in elderly patients and often lead to functional impairment. This study compared closed reduction with external fixation (EF) and open reduction with locking plate fixation (LP) in elderly patients. A retrospective analysis was conducted on 80 elderly patients with unilateral distal radius fractures treated in our hospital from 2021 to 2024. Patients were divided into a closed reduction and EF group (n = 40) and an open reduction and locking plate internal fixation group (n = 40), according to the surgical procedure. General demographic and fracture-related data were collected and compared between the 2 groups. Perioperative parameters, including operation time, perioperative hemoglobin changes (ΔHb), Hb decrease (ΔHb), and postoperative hospital stay, were evaluated. Perioperative outcomes, complications, Disabilities of the Arm, Shoulder and Hand (DASH) scores, wrist range of motion (ROM), and visual analogue scale (VAS) pain scores were compared. Baseline characteristics were comparable between the 2 groups. In terms of surgical parameters, the EF group demonstrated significantly shorter operative time, shorter postoperative hospital stay, and smaller perioperative hemoglobin decrease (all *P* < .001). Functionally, DASH scores at 3 and 6 months postoperatively were significantly lower in the locking plate group than in the EF group, and the excellent/good functional rate at 6 months was 85.0% versus 62.5% (*P* < .05). Wrist ROM improved over time in both groups, but the locking plate group showed better motion in all directions at 3 and 6 months, as well as a higher excellent ROM rate at 6 months (82.5% vs 65.0%, *P* < .05). Regarding pain, early postoperative VAS scores were similar between groups; from 1 month onward, VAS scores remained slightly lower in the locking plate group (*P* < .01). EF offers advantages of shorter surgery, less perioperative trauma, and faster recovery, making it suitable for patients with limited surgical tolerance. LP provides better functional recovery, wrist mobility, and long-term pain relief, and may be preferred for elderly patients with higher functional demands. Individualized treatment should be based on patient condition and functional expectations.

## 1. Introduction

Distal radius fractures are among the most common osteoporotic fractures in elderly individuals and are associated with substantial functional impairment and reduced quality of life.^[1-4]^ With the increasing aging population worldwide and the growing prevalence of osteoporosis, optimizing treatment strategies for elderly patients with distal radius fractures has become an important clinical challenge.^[5,6]^

Closed reduction with external fixation (EF) and open reduction with locking plate internal fixation are 2 widely used surgical approaches for the management of distal radius fractures. EF offers the advantages of shorter operative time, lower surgical trauma, and reduced perioperative burden, making it a valuable option for elderly patients with limited surgical tolerance.^[7-9]^ In contrast, locking plate fixation provides stable fracture fixation and restoration of anatomical alignment, thereby facilitating earlier functional rehabilitation and potentially improving wrist function.^[10-13]^ However, current evidence remains controversial regarding the relative benefits of these procedures in elderly patients. While several studies have reported superior radiographic and functional outcomes following locking plate fixation, others have suggested that EF can achieve acceptable outcomes in patients with limited functional demands and higher surgical risk.^[14-18]^

Furthermore, most previous studies have focused on isolated outcomes such as radiographic parameters, complication rates, or functional scores, whereas comprehensive evaluations integrating perioperative safety, complications, wrist mobility, and pain outcomes remain limited.^[19-23]^ Consequently, the optimal surgical strategy for elderly patients with distal radius fractures remains unclear, particularly when balancing surgical invasiveness against functional recovery.

Therefore, the present study retrospectively compared closed reduction with EF and open reduction with locking plate internal fixation in elderly patients with distal radius fractures. By simultaneously evaluating perioperative indicators, complications, functional recovery, wrist ROM, and pain outcomes, we aimed to provide evidence to support individualized surgical decision-makingand optimize treatment selection in this patient population.

## 2. Materials and methods

### 2.1. Study population

This study was approved by the Ethics Committee of Yichang Cent Peoples Hospital. This single-center retrospective comparative study enrolled elderly patients with unilateral distal radius fractures who underwent surgical treatment at our hospital between 2021 and 2024.

Inclusion criteria were:

(1) age ≥ 60 years;
(2) first-onset, unilateral, fresh distal radius fracture meeting radiographic diagnostic criteria;
(3) eligibility for surgical treatment with either closed reduction and EF or open reduction and internal fixation using a locking plate;
(4) complete preoperative imaging and clinical data, and ability to comply with follow-up and functional assessment.

Exclusion criteria were:

(1) concomitant severe fractures or neurovascular injuries involving other regions of the ipsilateral upper limb;
(2) pathological fractures or old (chronic) fractures;
(3) preexisting severe dysfunction or arthritis of the ipsilateral wrist;
(4) severe cardiopulmonary insufficiency, malignancy, psychiatric disorders, or other conditions rendering the patient unable to tolerate surgery or complete follow-up.

According to the actual surgical procedure received, patients were divided into 2 groups: a closed reduction and EF group and an open reduction with locking plate internal fixation group. Preoperative general data for both groups were extracted in a standardized manner from the medical record system by the same research team, including age, sex, height, weight, body mass index (BMI), smoking history, systolic blood pressure, diastolic blood pressure, heart rate, as well as fracture type (Colles, Smith, Barton, etc), mechanism of injury (low-energy fall, traffic accident, etc), and comorbidities such as hypertension and diabetes. These variables were used for baseline characteristic comparison and control of potential confounders.

The study protocol was approved by the institutional ethics committee and conducted in accordance with the principles of the Declaration of Helsinki. Written informed consent was obtained from all enrolled patients or their legal guardians.

### 2.2. Grouping and surgical procedures

#### 2.2.1. Closed reduction and external fixation group

Under brachial plexus block or local anesthesia, the patient was placed in the supine position with the affected limb positioned on a radiolucent table. Under C-arm fluoroscopic guidance, closed reduction was performed by the same orthopedic surgical team using a combination of sustained traction and maneuvers in palmar flexion/dorsal extension and ulnar/radial deviation according to the fracture pattern. Satisfactory reduction was defined as restoration of radiographic parameters such as volar tilt, radial inclination, and radial shortening of the wrist within clinically acceptable ranges. Immediately after satisfactory reduction, a plaster splint or cast was applied for EF, encompassing the distal forearm and wrist, with the wrist maintained in a functional position or slight extension. Fluoroscopy was repeated after casting to confirm the reduction and fixation position. The start and end times of the procedure were recorded intraoperatively to calculate operation time.

#### 2.2.2. Open reduction and internal fixation with locking plate group

Under brachial plexus block or general anesthesia, the patient was placed in the supine position with the affected upper limb on a hand table. A volar Henry approach or modified volar approach was used to expose the distal radius fracture. Open anatomical reduction was achieved using traction, levering, and bone-holding forceps, with temporary fixation using Kirschner wires when necessary. After satisfactory reduction was confirmed by C-arm fluoroscopy, a precontoured volar locking plate was implanted. Locking screws or cortical screws were selected according to the manufacturer’s instructions and the fracture configuration, with particular care taken to protect the flexor tendons and the median nerve. Final fluoroscopy was performed to confirm the position of the plate and screws as well as the quality of reduction, followed by thorough irrigation of the wound, layered closure of the incision, and compressive dressing. Operation time was recorded.

### 2.3. Perioperative management and rehabilitation protocol

In both groups, routine preoperative evaluations were completed, including a complete blood count, serum biochemistry, electrocardiography, and optimization of internal medical comorbidities. Anesthesia assessment was performed within 24 hours before surgery. Standard perioperative management, including prophylactic antibiotics, analgesia, and symptomatic supportive treatment, was administered intraoperatively and postoperatively.

Complete blood count was repeated preoperatively and on postoperative day 1 to measure hemoglobin (Hb) levels, which were used to assess perioperative blood loss. The change in hemoglobin (ΔHb) was calculated as ΔHb = preoperative Hb − postoperative day 1 Hb. Postoperative mobilization out of bed and initiation of functional exercises were determined based on the patient’s general condition and fracture stability.

In the closed reduction and EF group, patients were guided in the early postoperative period to perform active movements of the fingers, elbow, and shoulder, and wrist motion was gradually introduced once fracture stability was deemed adequate. In the open reduction and internal fixation with locking plate group, active and passive wrist exercises could be initiated earlier, provided that internal fixation was stable. The length of postoperative hospital stay was determined by the attending physician based on overall recovery, wound healing, and radiographic follow-up findings.

### 2.4. Outcome measures

#### 2.4.1. Baseline data

Preoperative demographic and clinical data were collected and organized for both groups, including age, sex, height, weight, BMI, smoking history, systolic blood pressure, diastolic blood pressure, heart rate, fracture type, mechanism of injury, and major comorbidities such as hypertension and diabetes. These variables were used for baseline characteristic analysis and to assess comparability between the 2 groups.

#### 2.4.2. Perioperative parameters

The main perioperative outcome measures included:

(1) Operation time: measured in minutes, from the start of skin preparation and draping to completion of external or internal fixation;
(2) Preoperative Hb and postoperative day 1 Hb: venous blood samples were obtained on the day before surgery and within 24 hours after surgery to measure hemoglobin (Hb);
(3) Decrease in Hb (ΔHb): calculated as preoperative Hb minus postoperative day 1 Hb;
(4) Postoperative hospital stay: recorded in days, from the first day after surgery to the day of discharge.

#### 2.4.3. Postoperative complications

All complications related to the surgical procedure and to external or internal fixation were recorded for both groups during follow-up. Complications were categorized into 3 types:

(1) infection-related complications: including superficial wound infection, judged based on clinical signs and symptoms and, when necessary, microbiological examination;
(2) fixator/plate-related complications: including local skin erythema or pressure sores caused by cast compression, and local pain or tendon irritation induced by plates or screws;
(3) fracture-healing–related complications: including malunion, delayed union, or nonunion, determined by a combination of radiographic findings and clinical criteria for fracture healing.

#### 2.4.4. Assessment of upper limb and wrist function

At 3 and 6 months postoperatively, upper limb and wrist function were evaluated using the Disabilities of the Arm, Shoulder and Hand (DASH) questionnaire. Patients completed the questionnaire under the guidance of assessors who had received standardized training. DASH scores were calculated according to the standard method, ranging from 0 to 100 points, with higher scores indicating more severe upper limb disability. Based on the DASH score at 6 months, a score <30 points was defined as “excellent/good” function and a score ≥30 points as “fair/poor,” and the excellent/good functional rate was calculated.

At the same time points (3 and 6 months postoperatively), a goniometer was used to measure the range of motion (ROM) of the affected wrist in all directions, including extension, flexion, ulnar deviation, and radial deviation, and all values were recorded in degrees (°). To further comprehensively evaluate wrist mobility, “excellent wrist range of motion” was defined as motion in all directions of the affected wrist reaching ≥80% of that of the contralateral side at 6 months postoperatively, and the excellent ROM rate was compared between the 2 groups.

#### 2.4.5. Pain assessment

At postoperative day 3, day 7, and at 1, 3, and 6 months after surgery, wrist pain intensity in both groups was assessed using the visual analogue scale (VAS). A 0 to 10 linear scale was used, where 0 indicated no pain and 10 indicated unbearable severe pain. Patients were asked to mark their current pain level on the scale, and the corresponding numerical value was recorded.

### 2.5. Follow-up methods

All patients were followed up postoperatively at predefined time points through outpatient visits or telephone interviews. At 1, 3, and 6 months after surgery, patients were scheduled to return for radiographic examination (X-ray) and completion of the DASH questionnaire, wrist ROM measurements, and VAS pain assessment. For patients with limited mobility, telephone follow-up was supplemented by caregiver-reported pain information, and efforts were made to arrange hospital visits at key time points to complete imaging and functional evaluations. The follow-up process was conducted by designated members of the research team to ensure consistency in assessment methods.

### 2.6. Statistical analysis

All data were entered into a database independently by 2 researchers using a double-entry method, followed by logical consistency checks. Continuous variables were expressed as mean ± standard deviation (*x̄* ± *s*), and between-group comparisons were performed using the independent-samples *t* test. Categorical variables were expressed as counts and percentages (n, %), and between-group comparisons were conducted using the χ^2^ test or Fisher’s exact test, as appropriate. A 2-sided *P* value < .05 was considered statistically significant. Statistical analyses were performed using SPSS or similar statistical software (e.g., SPSS version 26.0, IBM Corp.).

Although this was a retrospective study, a post hoc sample size estimation was performed based on the primary outcome of the 6-month DASH score. According to previous literature and preliminary clinical observations, a between-group difference of 6 points in DASH score was considered clinically meaningful, with an estimated standard deviation of 9 points. Using a 2-sided α level of 0.05 and a statistical power of 80% (β = 0.20), the minimum required sample size was calculated to be 36 patients per group. Therefore, the inclusion of 40 patients in each group (total n = 80) provided adequate statistical power to detect clinically relevant differences between treatment groups.

## 3. Results

### 3.1. Comparison of baseline characteristics

A total of 80 elderly patients with unilateral distal radius fractures who underwent surgical treatment at our hospital between 2021 and 2024 were included in this study. According to the type of surgical procedure, patients were divided into a closed reduction and EF group (n = 40) and an open reduction with locking plate internal fixation group (n = 40). There were no statistically significant differences between the 2 groups in general demographic characteristics, anthropometric parameters, or comorbidities (*P* > .05), indicating good baseline comparability. The mean age was (74.5 ± 6.2) years in the closed reduction and EF group and (75.0 ± 5.9) years in the open reduction with locking plate group (*P* = .812). Regarding sex distribution, there were 17 males (42.5%) and 23 females (57.5%) in the former, and 18 males (45.0%) and 22 females (55.0%) in the latter (*P* = .874).

In terms of anthropometric parameters, the mean height was (163.4 ± 7.1) cm versus (162.8 ± 6.9) cm (*P* = .702), and the mean weight was (62.7 ± 8.6) kg versus (63.2 ± 8.1) kg (*P* = .812) in the closed reduction and EF group and the locking plate group, respectively. The corresponding BMI values were (23.5 ± 2.9) kg/m^2^ and (23.8 ± 2.7) kg/m^2^ (*P* = .613). With respect to lifestyle factors, the number of patients with a history of smoking was 8 (20.0%) and 9 (22.5%) in the 2 groups, respectively (*P* = .784).

Comparison of vital signs at admission showed no significant differences between the 2 groups: systolic blood pressure was (136.8 ± 14.9) mm Hg versus (138.2 ± 15.1) mm Hg (*P* = .679), diastolic blood pressure was (78.6 ± 9.2) mm Hg versus (79.4 ± 8.7) mm Hg (*P* = .687), and heart rate was (76.9 ± 8.6) beats/min versus (77.6 ± 8.1) beats/min (*P* = .711) in the closed reduction and EF group and the locking plate group, respectively.

With regard to fracture-related characteristics, the distribution of fracture types was similar between groups. In the closed reduction and EF group, there were 33 Colles fractures (82.5%), 5 Smith fractures (12.5%), and 2 Barton fractures (5.0%), while in the locking plate group there were 32 (80.0%), 6 (15.0%), and 2 (5.0%) cases, respectively (*P* = .932). Low-energy falls were the predominant mechanism of injury: in the closed reduction and EF group, 27 patients (67.5%) were injured by falls and 13 (32.5%) by traffic accidents; in the locking plate group, 26 (65.0%) and 14 (35.0%) patients sustained injuries due to falls and traffic accidents, respectively (*P* = .786).

Regarding common comorbidities, the proportions of patients with hypertension were 12 (30.0%) and 13 (32.5%), and those with diabetes mellitus were 10 (25.0%) and 11 (27.5%) in the 2 groups, respectively, with no significant differences (*P* = .842). In summary, the 2 groups were balanced and comparable in terms of baseline characteristics including age, sex, height, weight, BMI, smoking history, blood pressure, heart rate, fracture type, mechanism of injury, and major comorbidities (Table 1).

**Table 1:** Comparison of baseline characteristics between the 2 groups.

Variable	Closed reduction and external fixation group (n = 40)	Open reduction and locking plate internal fixation group (n = 40)	Statistic	*P* value
Age (yr, mean ± SD)	74.5 ± 6.2	75.0 ± 5.9	*t* = 0.24	.812
Sex (male/female, n, %)	17 (42.5)/23 (57.5)	18 (45.0)/22 (55.0)	χ^2^ = 0.03	.874
Height (cm, mean ± SD)	163.4 ± 7.1	162.8 ± 6.9	*t* = 0.38	.702
Weight (kg, mean ± SD)	62.7 ± 8.6	63.2 ± 8.1	*t* = 0.24	.812
BMI (kg/m^2^, mean ± SD)	23.5 ± 2.9	23.8 ± 2.7	*t* = 0.51	.613
Smoking history (n, %)	8 (20.0)	9 (22.5)	χ^2^ = 0.08	.784
Systolic blood pressure (SBP, mm Hg, mean ± SD)	136.8 ± 14.9	138.2 ± 15.1	*t* = 0.42	.679
Diastolic blood pressure (DBP, mm Hg, mean ± SD)	78.6 ± 9.2	79.4 ± 8.7	*t* = 0.41	.687
Heart rate (beats/min, mean ± SD)	76.9 ± 8.6	77.6 ± 8.1	*t* = 0.37	.711
Fracture type (Colles/Smith/Barton, n, %)	33 (82.5)/5 (12.5)/2 (5.0)	32 (80.0)/6 (15.0)/2 (5.0)	χ^2^ = 0.14	.932
Mechanism of injury (fall/traffic accident, n, %)	27 (67.5)/13 (32.5)	26 (65.0)/14 (35.0)	χ^2^ = 0.07	.786
Hypertension (n, %)	12 (30.0)	13 (32.5)	χ^2^ = 0.04	.842
Diabetes mellitus (n, %)	10 (25.0)	11 (27.5)	χ^2^ = 0.05	.842

BMI = body mass index, DBP = diastolic blood pressure, SBP = systolic blood pressure, SD = standard deviation.

### 3.2. Comparison of operation time and perioperative hemoglobin-related parameters

All patients in both groups successfully completed surgical treatment, and no perioperative deaths occurred. Comparison of surgical parameters showed that operation time was significantly shorter in the closed reduction and EF group than in the open reduction with locking plate internal fixation group, with values of (20.8 ± 6.5) min and (69.2 ± 10.3) min, respectively (*t* = 25.13, *P* < .001). Since closed reduction with EF does not involve an open approach, intraoperative blood loss could not be accurately quantified; therefore, changes in perioperative hemoglobin levels were used as an indirect indicator of blood loss.

Preoperatively, there was a certain difference in hemoglobin levels between the 2 groups: (129.6 ± 13.5) g/L in the closed reduction and EF group versus (123.8 ± 12.9) g/L in the locking plate group; however, this difference was not statistically significant (*t* = 1.91, *P* = .060). On postoperative day 1, hemoglobin levels had decreased in both groups compared with preoperative values, being (125.1 ± 13.1) g/L in the closed reduction and EF group and (112.6 ± 13.4) g/L in the locking plate group, with a statistically significant between-group difference (*t* = 3.88, *P* < .001).

Further analysis of the decrease in hemoglobin (ΔHb) showed values of (4.5 ± 2.6) g/L in the closed reduction and EF group and (11.2 ± 3.8) g/L in the locking plate group, indicating a markedly greater decline in the latter (*t* = 9.02, *P* < .001).

With respect to postoperative hospital stay, the duration was (4.0 ± 1.2) days in the closed reduction and EF group, significantly shorter than (6.3 ± 1.6) days in the locking plate group (*t* = 7.28, *P* < .001).

**Figure 1.**
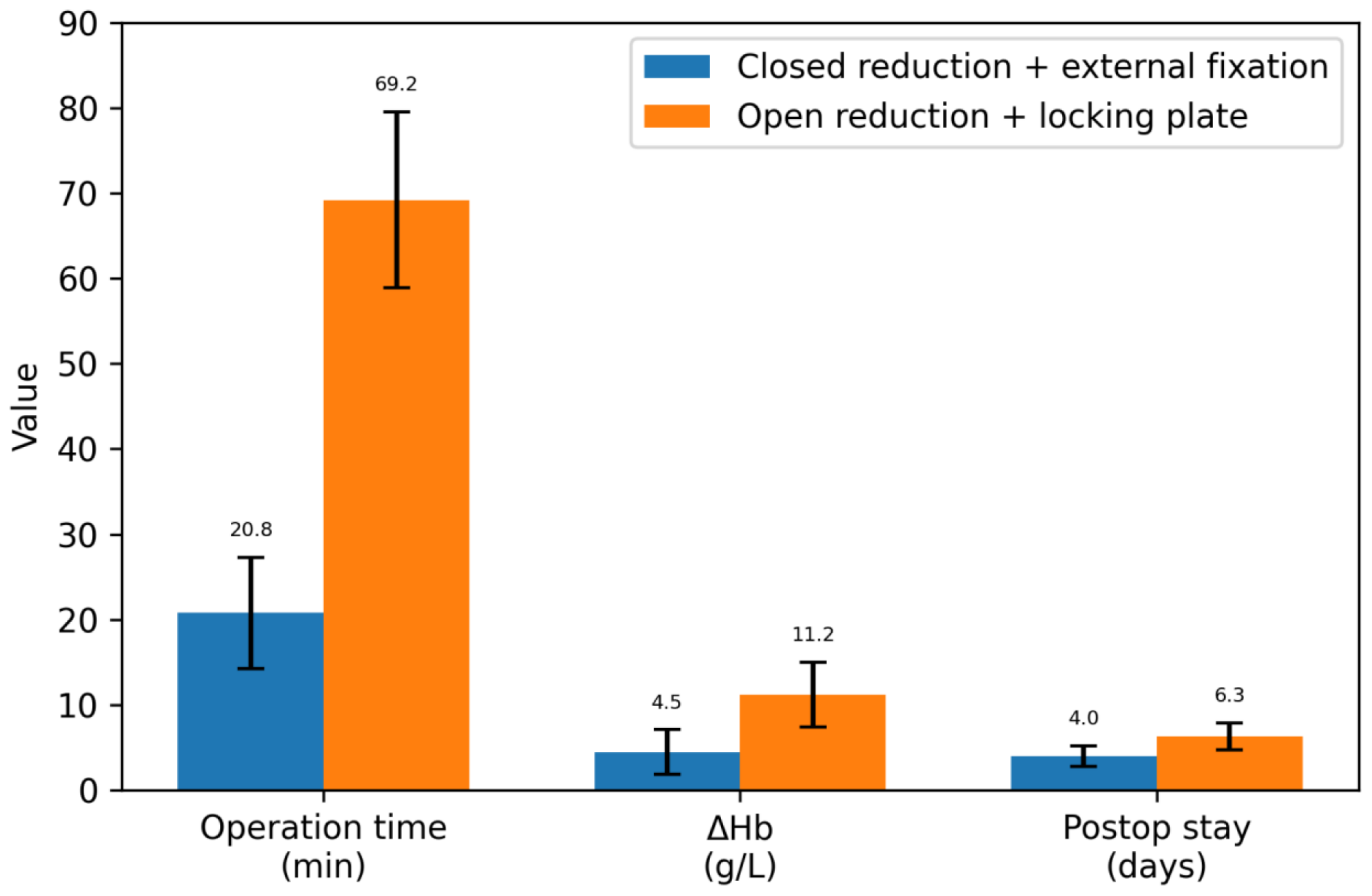
Comparison of operative time, ΔHb, and postoperative hospital stay between the 2 groups. ΔHb = Delta Hemoglobin (Change in Hemoglobin).

These findings suggest that, under the premise of surgical safety, closed reduction with EF has clear advantages over open reduction with locking plate internal fixation in terms of shortening operation time, reducing perioperative hemoglobin decline associated with blood loss, and decreasing postoperative length of hospital stay. Details are shown in Table 2 and Figure 1.

**Table 2:** Comparison of operative time and perioperative parameters between the 2 groups.

Variable	Closed reduction and external fixation group (n = 40)	Open reduction and locking plate internal fixation group (n = 40)	Statistic	*P* value
Operation time (min, mean ± SD)	20.8 ± 6.5	69.2 ± 10.3	*t* = 25.13	<.001
Preoperative Hb (g/L, mean ± SD)	129.6 ± 13.5	123.8 ± 12.9	*t* = 1.91	.060
Postoperative day 1 Hb (g/L, mean ± SD)	125.1 ± 13.1	112.6 ± 13.4	*t* = 3.88	<.001
Decrease in Hb (ΔHb, g/L, mean ± SD)	4.5 ± 2.6	11.2 ± 3.8	*t* = 9.02	<.001
Postoperative hospital stay (d, mean ± SD)	4.0 ± 1.2	6.3 ± 1.6	*t* = 7.28	<.001

ΔHb = Delta Hemoglobin (Change in Hemoglobin), SD = standard deviation.

### 3.3. Comparison of postoperative complication rates

No patients in either group developed severe complications such as compartment syndrome, deep infection, or severe nonunion. The overall incidence of postoperative complications was 10.0% (4/40) in the closed reduction and EF group and 15.0% (6/40) in the open reduction with locking plate internal fixation group, with no statistically significant difference between the 2 groups (χ^2^ = 0.46, *P* = .499).

In this study, closed reduction and EF were mainly achieved using plaster splints or braces. The complications in this group were predominantly cast-related and fracture-healing–related issues: 2 patients (5.0%) developed local skin erythema or mild pressure sores due to cast compression, all of which improved after timely adjustment or replacement of the cast; 2 patients (5.0%) experienced mild malunion due to suboptimal maintenance of reduction, which manifested only as slight angulation on imaging and had limited impact on wrist function.

In the open reduction with locking plate internal fixation group, complications were mainly related to wound healing and the internal fixation device. Superficial wound infection occurred in 1 patient (2.5%) and resolved after local wound care and a short course of antibiotic therapy. Plate or screw irritation led to local pain or tendon irritation in 3 patients (7.5%); among them, 1 patient underwent implant removal after fracture union, with marked symptom relief. Delayed fracture union was observed in 2 patients (5.0%), both of whom ultimately achieved bony union after prolonged follow-up and continued functional exercises.

Stratified analysis by complication type showed that infection-related complications occurred in 0 patients (0%) in the closed reduction and EF group and in 1 patient (2.5%) in the locking plate group, with no statistically significant difference (Fisher’s exact test, *P* = .314). Fixator/plate-related complications were observed in 2 patients (5.0%) versus 3 patients (7.5%) in the 2 groups, respectively (χ^2^ = 0.22, *P* = .640). Fracture-healing–related complications were seen in 2 patients (5.0%) in each group (χ^2^ = 0.00, *P* = 1.000).

**Figure 2.**
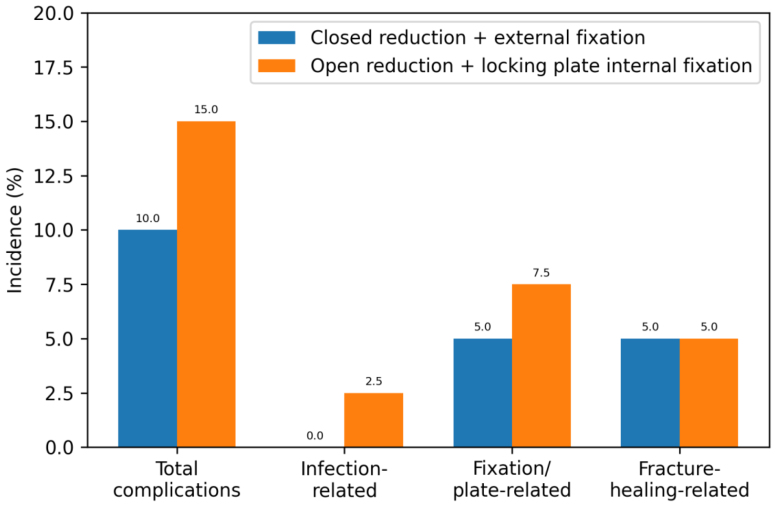
Incidence of complications after closed reduction with external fixation and ORIF with locking plate. ORIF = Open Reduction and Internal Fixation.

Overall, both surgical techniques were associated with a low incidence of postoperative complications and demonstrated good safety profiles in elderly patients with distal radius fractures. Although the locking plate group showed a slightly higher frequency of implant-related soft tissue irritation, this did not result in a significant adverse impact on final fracture union or wrist function (Table 3, Fig. 2).

**Table 3:** Postoperative complications in the 2 groups.

Variable	Closed reduction and external fixation group (n = 40)	Open reduction and locking plate internal fixation group (n = 40)	Statistic	*P* value
Total complications (n, %)	4 (10.0)	6 (15.0)	χ^2^ = 0.46	.499
Infection-related complications(n, %)	0 (0)	1 (2.5)	Fisher	.314
Fixation/plate-related complications (n, %)	2 (5.0)	3 (7.5)	χ^2^ = 0.22	.640
Fracture-healing–related complications(n, %)	2 (5.0)	2 (5.0)	χ^2^ = 0.00	1.000

### 3.4. Comparison of postoperative functional recovery

In this study, postoperative upper limb and wrist function was evaluated in both groups using the Disabilities of the Arm, Shoulder and Hand (DASH) questionnaire, with lower DASH scores indicating better function. At 3 months postoperatively, the mean DASH score was (46.3 ± 9.1) points in the closed reduction and EF group and (37.8 ± 8.4) points in the open reduction with locking plate internal fixation group, with a statistically significant difference between the 2 groups (*t* = 4.34, *P* < .001), suggesting that early functional recovery was superior in the locking plate group.

By 6 months postoperatively, functional scores in both groups had improved markedly compared with those at 3 months; however, the open reduction with locking plate group continued to demonstrate an advantage. The mean DASH scores at 6 months were (35.2 ± 8.5) points in the closed reduction and EF group and (28.6 ± 6.3) points in the locking plate group, and the difference remained statistically significant (*t* = 3.95, *P* < .001).

Using a DASH score <30 points at 6 months postoperatively to define “excellent/good” function and ≥30 points to define “fair/poor,” the excellent/good functional rates were 62.5% (25/40) in the closed reduction and EF group and 85.0% (34/40) in the locking plate group. The difference in excellent/good rates between the 2 groups was statistically significant (χ^2^ = 5.23, *P* = .022).

**Figure 3.**
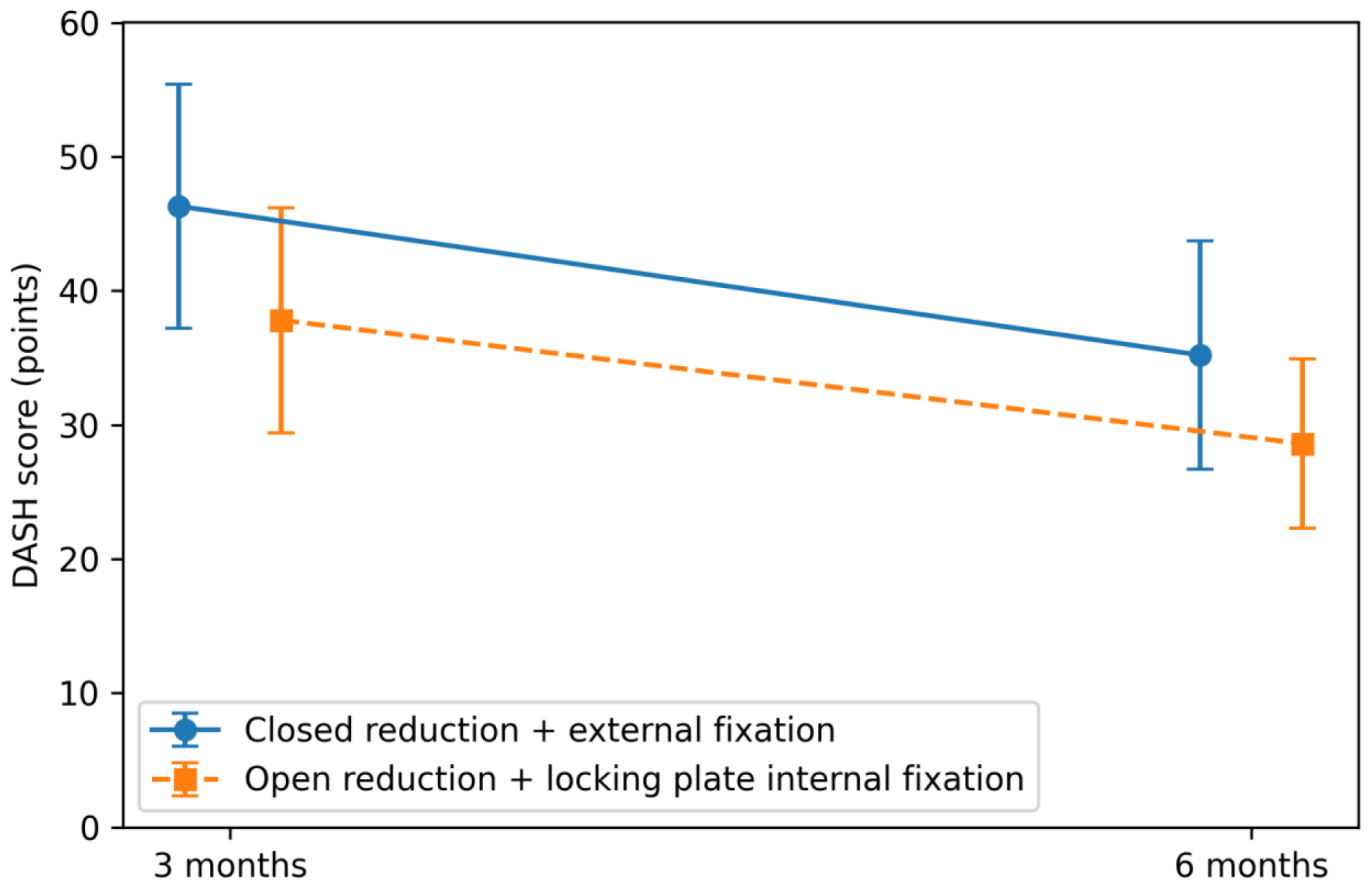
DASH scores at 3 and 6 months postoperatively in the 2 treatment groups. DASH = Disabilities of the Arm, Shoulder and Hand.

These findings indicate that although both surgical techniques can significantly improve upper limb and wrist function in elderly patients with distal radius fractures over the follow-up period, open reduction with locking plate internal fixation is superior to closed reduction with EF in terms of both the speed of functional recovery and the final excellent/good functional rate (Table 4, Fig. 3).

**Table 4:** Comparison of postoperative functional recovery between the 2 groups.

Variable	Closed reduction and external fixation group (n = 40)	Open reduction and locking plate internal fixation group (n = 40)	Statistic	*P* value
DASH score at 3 mo postoperatively (points, mean ± SD)	46.3 ± 9.1	37.8 ± 8.4	*t* = 4.34	<.001
DASH score at 6 mo postoperatively (points, mean ± SD)	35.2 ± 8.5	28.6 ± 6.3	*t* = 3.95	<.001
Excellent/good function at 6 mo (n, %)	25 (62.5)	34 (85.0)	χ^2^ = 5.23	.022

DASH = Disabilities of the Arm, Shoulder and Hand, SD = standard deviation.

### 3.5. Comparison of postoperative wrist range of motion

In this study, a goniometer was used to objectively measure wrist ROM of the affected side in both groups at 3 and 6 months postoperatively, including extension, flexion, ulnar deviation, and radial deviation. At 3 months after surgery, recovery of wrist ROM was relatively slower in the closed reduction and EF group, with extension, flexion, ulnar deviation, and radial deviation of (50.2 ± 7.8)°, (45.1 ± 6.9)°, (20.3 ± 4.1)°, and (25.4 ± 4.8)°, respectively. In the open reduction with locking plate internal fixation group, the corresponding values were (55.3 ± 7.1)°, (49.8 ± 6.2)°, (22.1 ± 3.5)°, and (28.3 ± 4.2)°, all of which were higher than those in the closed reduction and EF group. The differences in all 4 directions of motion were statistically significant (*t* = 2.16–3.12, all *P* < .05), indicating better early recovery of wrist ROM in the internal fixation group.

By 6 months postoperatively, wrist ROM in both groups had improved markedly compared with that at 3 months. In the closed reduction and EF group, extension, flexion, ulnar deviation, and radial deviation reached (60.1 ± 7.2)°, (55.6 ± 6.4)°, (25.2 ± 4.0)°, and (30.5 ± 4.6)°, respectively, while the corresponding values in the locking plate group were (64.5 ± 6.3)°, (59.3 ± 5.8)°, (27.4 ± 3.6)°, and (32.9 ± 4.1)°. The between-group differences remained statistically significant (*t* = 2.37–2.69, all *P* < .05), although the gap was slightly smaller than that observed at 3 months.

When “excellent wrist ROM” was defined as ROM in all directions of the affected wrist reaching ≥80% of that of the contralateral side at 6 months postoperatively, the excellent ROM rate was 65.0% (26/40) in the closed reduction and EF group and 82.5% (33/40) in the locking plate group, with a statistically significant difference between the 2 groups (χ^2^ = 4.02, *P* = .045).

**Figure 4.**
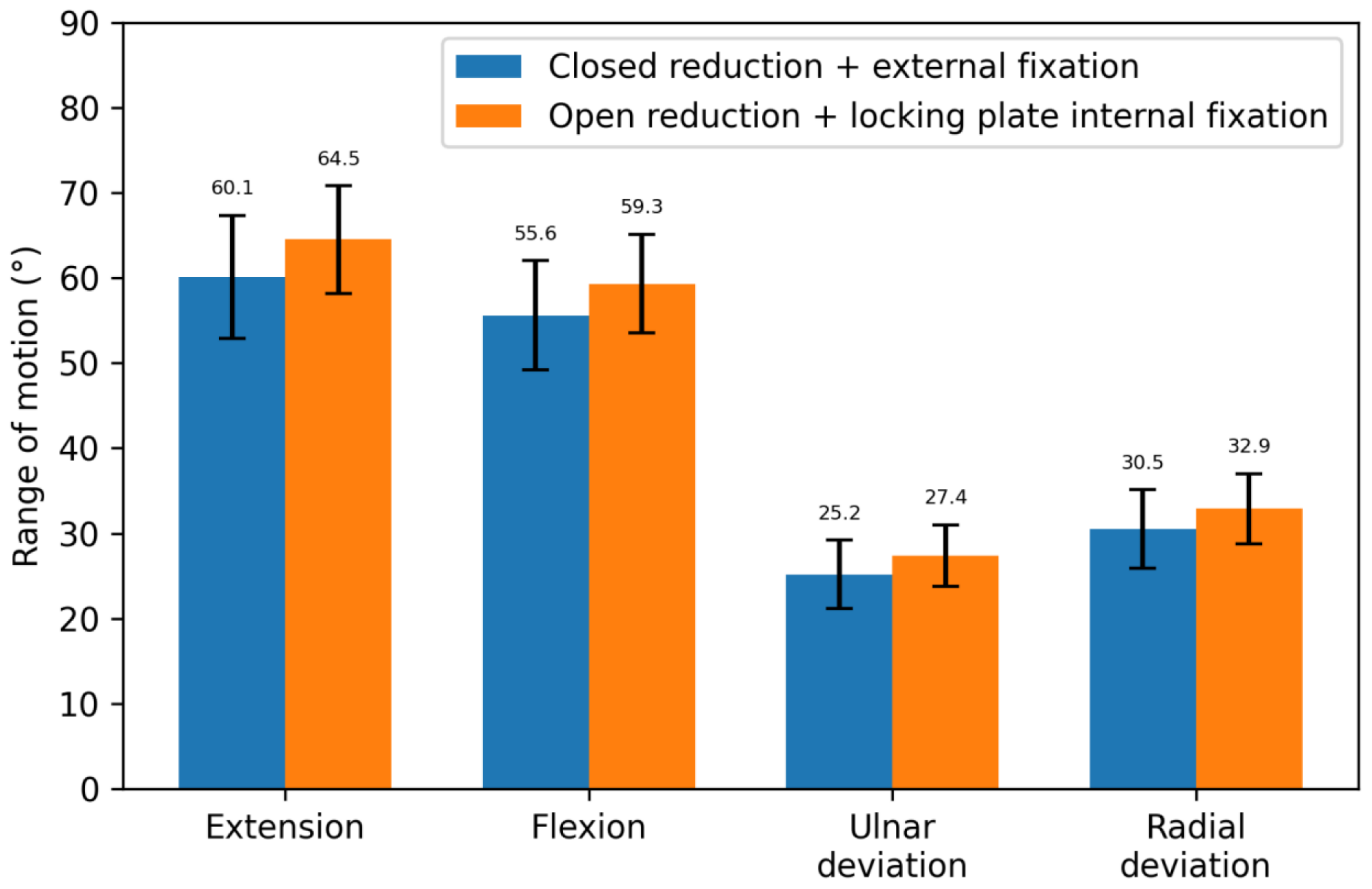
Wrist range of motion at 6 months postoperatively in the 2 treatment groups.

In summary, both surgical techniques led to significant improvement in wrist ROM over time in elderly patients with distal radius fractures; however, open reduction with locking plate internal fixation resulted in superior recovery of wrist ROM at both early and mid-term follow-up compared with closed reduction and EF (Table 5, Fig. 4).

**Table 5:** Comparison of postoperative wrist range of motion between the 2 groups.

Variable	Closed reduction and external fixation group (n = 40)	Open reduction and locking plate internal fixation group (n = 40)	Statistic	*P* value
Wrist extension at 3 mo postoperatively (°, mean ± SD)	50.2 ± 7.8	55.3 ± 7.1	*t* = 3.12	.003
Wrist flexion at 3 mo postoperatively (°, mean ± SD)	45.1 ± 6.9	49.8 ± 6.2	*t* = 3.01	.004
Ulnar deviation at 3 mo postoperatively (°, mean ± SD)	20.3 ± 4.1	22.1 ± 3.5	*t* = 2.16	.034
Radial deviation at 3 mo postoperatively (°, mean ± SD)	25.4 ± 4.8	28.3 ± 4.2	*t* = 2.96	.004
Wrist extension at 6 mo postoperatively (°, mean ± SD)	60.1 ± 7.2	64.5 ± 6.3	*t* = 2.69	.009
Wrist flexion at 6 mo postoperatively (°, mean ± SD)	55.6 ± 6.4	59.3 ± 5.8	*t* = 2.61	.011
Ulnar deviation at 6 mo postoperatively (°, mean ± SD)	25.2 ± 4.0	27.4 ± 3.6	*t* = 2.53	.013
Radial deviation at 6 mo postoperatively (°, mean ± SD)	30.5 ± 4.6	32.9 ± 4.1	*t* = 2.37	.020
Excellent wrist range of motion at 6 mo (n, %)	26 (65.0)	33 (82.5)	χ^2^ = 4.02	.045

SD = standard deviation.

### 3.6. Comparison of postoperative pain assessment

In this study, wrist pain at different postoperative time points was evaluated in both groups using the visual analogue scale (VAS, 0–10 points, with higher scores indicating more severe pain). The results showed that both groups experienced moderate pain in the early postoperative period (days 3 and 7), with no significant differences between them. On postoperative day 3, the VAS score was (6.8 ± 1.5) points in the closed reduction and EF group and (7.0 ± 1.6) points in the open reduction with locking plate internal fixation group (*t* = 0.58, *P* = .566). On postoperative day 7, the scores were (5.6 ± 1.4) points and (5.3 ± 1.5) points, respectively, again with no statistically significant difference (*t* = 0.92, *P* = .359).

From 1 month after surgery onward, pain intensity in both groups decreased markedly and was generally in the mild range, although VAS scores were consistently slightly lower in the locking plate group. At 1 month postoperatively, the VAS score was (3.2 ± 1.2) points in the closed reduction and EF group and (2.6 ± 1.0) points in the locking plate group, with a statistically significant difference (*t* = 2.43, *P* = .018). At 3 months, the scores further decreased to (2.5 ± 1.0) points and (1.9 ± 0.9) points, respectively (*t* = 2.82, *P* = .006). At 6 months postoperatively, the VAS scores were (2.1 ± 0.9) points in the closed reduction and EF group and (1.5 ± 0.8) points in the locking plate group (*t* = 3.15, *P* = .002).

**Figure 5.**
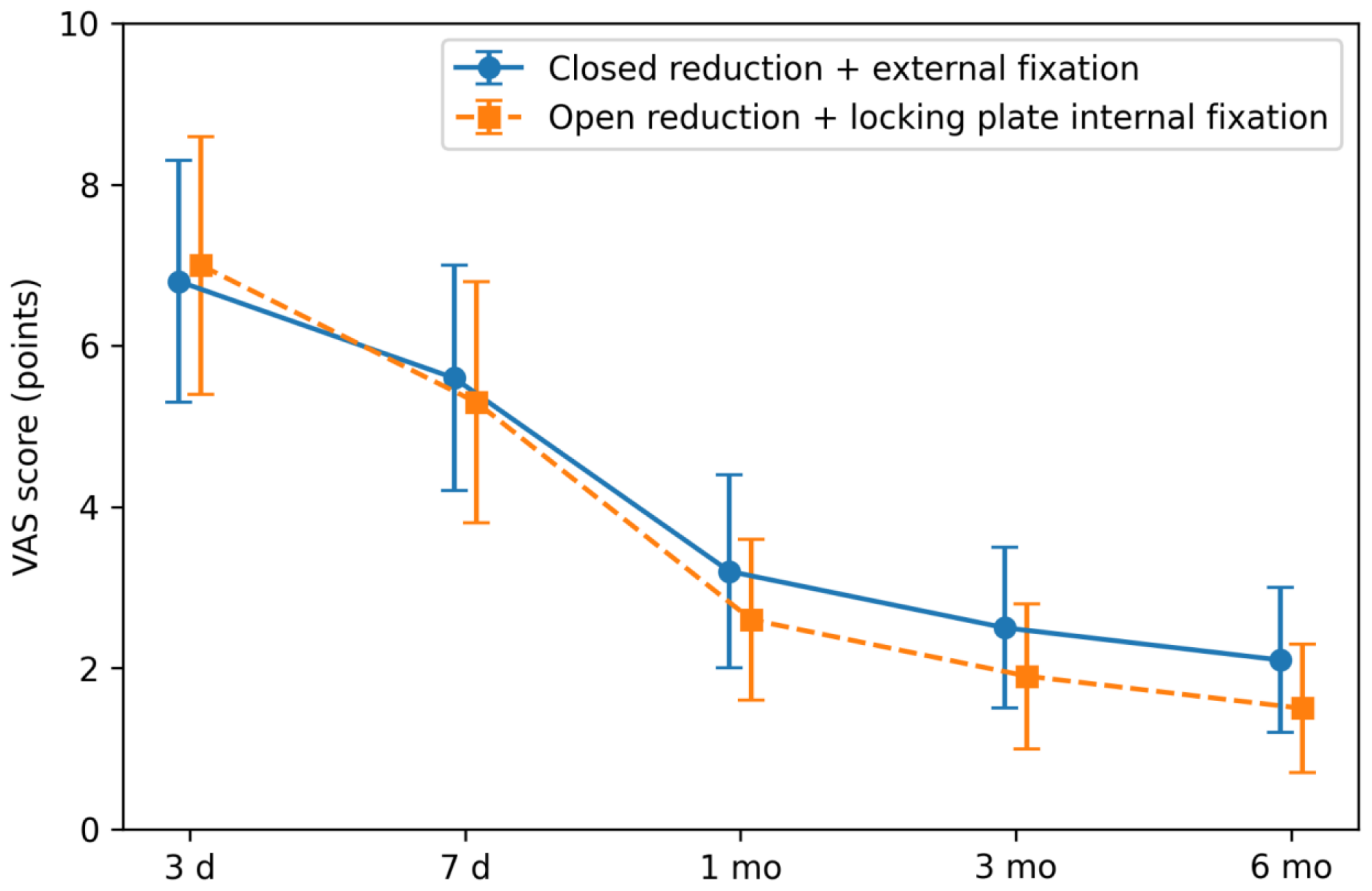
Changes in VAS pain scores over time in the 2 treatment groups. VAS = Visual Analogue Scale.

These findings indicate that both surgical techniques can significantly reduce postoperative pain over time in elderly patients with distal radius fractures, and that most patients experience only mild pain from 1 month postoperatively onward. However, starting from the 1-month follow-up, VAS scores at each time-point were consistently slightly lower in the open reduction with locking plate internal fixation group than in the closed reduction and EF group, suggesting a certain advantage of the former in mid- to long-term pain control (Table 6, Fig. 5).

**Table 6:** Comparison of postoperative pain (VAS) at different time points between the 2 groups.

Time-point	Closed reduction and external fixation group (n = 40), VAS (points, mean ± SD)	Open reduction and locking plate internal fixation group (n = 40), VAS (points, mean ± SD)	Statistic	*P* value
Postoperative day 3	6.8 ± 1.5	7.0 ± 1.6	*t* = 0.58	.566
Postoperative day 7	5.6 ± 1.4	5.3 ± 1.5	*t* = 0.92	.359
1 mo postoperatively	3.2 ± 1.2	2.6 ± 1.0	*t* = 2.43	.018
3 mo postoperatively	2.5 ± 1.0	1.9 ± 0.9	*t* = 2.82	.006
6 mo postoperatively	2.1 ± 0.9	1.5 ± 0.8	*t* = 3.15	.002

SD = standard deviation, VAS = Visual Analogue Scale.

## 4. Discussion

Distal radius fracture is one of the most common fractures in the elderly population and is closely associated with low-energy falls and osteoporosis.^[24]^ With increasing life expectancy and accelerating population aging, how to ensure treatment safety while simultaneously optimizing functional recovery and quality of life has become a central issue in the management of distal radius fractures in older adults. Closed reduction with EF and open reduction with volar locking plate fixation are the 2 most frequently used surgical options. The former is characterized by minimal invasiveness and technical simplicity, whereas the latter relies on anatomical reduction and stable internal fixation, which facilitates early functional exercise.^[25,26]^ However, there is still ongoing debate in clinical practice regarding the optimal choice of procedure for elderly patients, and the present study was conducted in this context.

In this study, elderly patients with unilateral distal radius fractures treated by the same orthopedic team during the same period were grouped according to a unified set of inclusion and exclusion criteria, and the 2 procedures were systematically compared in terms of perioperative parameters, complications, functional recovery, wrist ROM, and pain trajectories. Overall, the 2 groups were generally comparable in preoperative status, fracture pattern, and comorbidity burden, providing a reliable basis for interpreting subsequent differences in outcomes. Unlike previous studies that often focused on a single aspect of treatment, this study integrated multiple functional and physiological indicators, aiming to address, in a manner closer to real-world decision-making, the practical question of “which procedure is more appropriate for elderly patients.”

From a perioperative perspective, the advantages of closed reduction with EF lie mainly in its technical simplicity, shorter operation time, and shorter postoperative hospital stay, which align well with the clinical reality that elderly patients often have multiple systemic comorbidities and limited surgical tolerance. In contrast, locking plate fixation requires open reduction and implant placement, resulting in longer operation times and higher demands on anesthesia and perioperative management. An important methodological feature of this study is the use of perioperative hemoglobin changes, rather than subjective intraoperative estimation alone, to reflect blood loss. The findings suggest that while internal fixation provides more stable mechanical support, it does so at the expense of greater surgical “trauma,” which is consistent with the concept reported in the literature that “internal fixation is more invasive but overall controllable.” This also underscores the need for careful risk–benefit assessment when considering internal fixation in very elderly patients or those with poor medical reserve.

With respect to complications, the overall incidence was low in both groups, indicating that, under standardized surgical techniques and appropriate perioperative management, both closed reduction with EF and locking plate fixation are generally safe and feasible in elderly patients. Notably, the spectrum of complications differed between procedures: EF was mainly associated with cast-related skin problems and mild malunion, whereas internal fixation was more frequently associated with wound healing issues and plate or screw irritation. Similar patterns have been reported in previous studies, suggesting that EF requires particular attention to cast application quality and radiographic monitoring of reduction maintenance during follow-up, while internal fixation demands careful protection of soft tissues, precise implant positioning, and timely recognition and management of implant-related discomfort during follow-up. The absence of severe deep infection, compartment syndrome, or catastrophic complications in this cohort further supports the safety of both procedures when used within appropriate indications.

Regarding functional outcomes, this study employed the DASH scoring system to assess upper limb and wrist function and additionally introduced the “excellent/good functional rate” as a categorical indicator. The results showed that the locking plate group achieved better functional recovery than the EF group at both mid-term and slightly longer-term follow-up, which is consistent with multiple clinical studies reporting that locking plate fixation favors earlier mobilization and functional restoration.^[27,28]^ The likely mechanism is that volar locking plates reconstruct the distal radius anatomy and provide a more stable internal fixation environment, allowing patients to initiate more active wrist and upper limb exercises during the early stages of fracture healing. This, in turn, helps to reduce the incidence of joint stiffness and soft tissue contracture. In addition to comparing mean scores, the present study used an excellent/good threshold to enhance the clinical interpretability of functional results, thereby facilitating communication of expected outcomes between clinicians and patients.

Recovery of wrist ROM is also a key determinant of treatment success in elderly patients with distal radius fractures. In this study, objective measurements of extension, flexion, ulnar deviation, and radial deviation were obtained at 3 and 6 months postoperatively. The results demonstrated that both procedures led to significant improvements in wrist motion over time; however, the locking plate group achieved motion closer to that of the contralateral side in all directions, with between-group differences being particularly pronounced in the early postoperative period. These findings are highly consistent with the treatment concept emphasizing “stable internal fixation plus early functional exercise.” By defining “excellent” wrist motion as achieving a certain proportion of the contralateral side, the study evaluated clinical benefit from an overall functional perspective rather than focusing on isolated angular measurements alone.

In terms of pain control, the multi-time-point VAS assessment adopted in this study delineated the dynamic trajectory of postoperative pain. Both groups experienced considerable pain in the early postoperative phase, which gradually subsided, with overall pain levels being low from 1 month onward. The locking plate group exhibited slightly lower VAS scores at mid- to longer-term follow-up, which may be related to more stable fracture fixation and a lower incidence of malunion, whereas the EF group may bear a higher risk of loss of reduction and cast-related discomfort. Previous studies have suggested that anatomical reduction and rigid internal fixation contribute to mitigating chronic residual pain, and the present findings are broadly in line with this view. The multi-time-point design also clarified the temporal evolution of pain, representing another methodological strength of this study.

The main innovations of this study can be summarized in 3 aspects. First, for the specific population of elderly patients with distal radius fractures, a relatively comprehensive evaluation framework was established across 5 dimensions: perioperative parameters, complications, function, ROM, and pain, rather than limiting the analysis to a single endpoint. Second, in terms of outcome selection, the study adopted ΔHb, multi-time-point VAS scores, functional excellent/good rate, and motion excellent rate as clinically interpretable composite indicators, thereby making the results more directly applicable to clinical decision-making. Third, surgical and rehabilitation protocols were highly standardized for all patients, reducing variability arising from differences in surgeons and rehabilitation strategies. Compared with previous multicenter studies with substantial heterogeneity, this design is more conducive to isolating the intrinsic differences between the 2 procedures.^[29,30]^

Nevertheless, this study has some limitations. First, it was a single-center retrospective study with a relatively limited sample size, and its conclusions require validation by larger-scale prospective studies. Second, the follow-up duration was mainly limited to 6 months postoperatively and therefore does not fully capture long-term issues related to implants, degenerative changes, or late functional evolution. Third, the correlation between radiographic quality of reduction and functional outcomes was not systematically analyzed. Future studies are needed to integrate parameters such as volar tilt, radial inclination, and radial shortening to elucidate more clearly the relationship between anatomical reduction and functional recovery. ASA classification was not routinely recorded and therefore could not be included in subgroup analyses.

Overall, this study suggests that, in elderly patients with distal radius fractures, closed reduction with EF has advantages in terms of shorter operation time, shorter hospital stay, and more controlled surgical trauma, and may be more suitable for patients with higher surgical risk or relatively low functional demands. Open reduction with locking plate internal fixation, by contrast, offers superior upper limb functional recovery, better wrist ROM, and improved mid- to long-term pain control, making it more appropriate for elderly patients who can tolerate surgical trauma and have higher functional expectations. In clinical practice, individualized treatment strategies should be formulated based on a comprehensive assessment of the patient’s general condition, bone quality, functional goals, and economic capacity, with the aim of achieving an optimal balance between fracture healing and functional recovery.

## 5. Conclusion

Both closed reduction with EF and open reduction with locking plate internal fixation are safe and effective treatment options for distal radius fractures in elderly patients. However, each technique demonstrates distinct clinical advantages.

Closed reduction with EF is associated with shorter operative time, less perioperative blood loss, and shorter hospitalization, making it particularly suitable for elderly patients with multiple medical comorbidities, higher anesthetic risk (ASA III–IV), limited physiological reserve, or relatively low functional demands.

In contrast, open reduction with locking plate internal fixation provides superior recovery of upper limb function, greater wrist ROM, and better long-term pain control. Therefore, it may be the preferred option for patients with unstable or intra-articular comminuted fractures, good overall health status, and higher expectations for postoperative wrist function.

Clinical decision-making should be individualized according to fracture characteristics, patient comorbidity burden, surgical tolerance, and functional goals to achieve the optimal balance between procedural safety and functional recovery.

## Author contributions

**Conceptualization:** Weidong Chen, Yu Nie, Naxin Zhou.

**Data curation:** Weidong Chen, Yu Nie, Naxin Zhou.

**Formal analysis:** Weidong Chen, Yu Nie, Naxin Zhou.

**Funding acquisition:** Weidong Chen, Yu Nie, Naxin Zhou.

**Investigation:** Weidong Chen, Naxin Zhou.

**Writing – original draft:** Naxin Zhou.

**Writing – review & editing:** Naxin Zhou.
